# Supplementary material for: Complex‐centric proteome profiling by SEC‐SWATH‐MS
Source: Mol Syst Biol. 2019 Jan 14;15(1):e8438. doi: 10.15252/msb.20188438 (PMC6346213; doi:10.15252/msb.20188438)
Supplement: Supplementary file 7 — Dataset EV6 [file MSB-15-e8438-s007.zip › feature_plots_bioplex/Q86VR2.pdf]

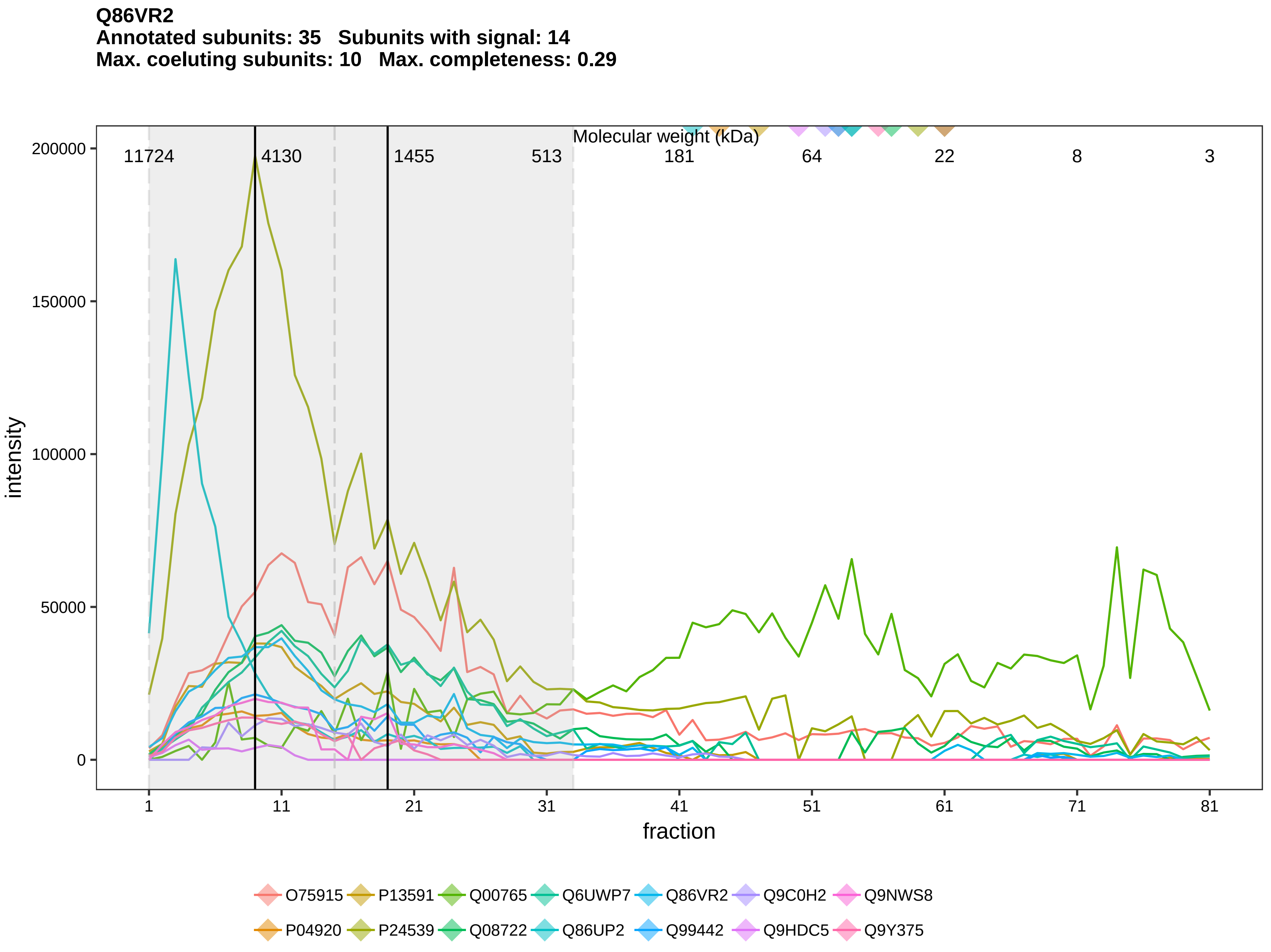

Q86VR2
Annotated subunits: 35 Subunits with signal: 14
Max. coeluting subunits: 10 Max. completeness: 0.29
Molecular weight (kDa)
11724
4130
1455
513
181
64
22
8
3
intensity
200000
150000
100000
50000
0
fraction
1
11
21
31
41
51
61
71
81
O75915
P13591
Q00765
Q6UWP7
Q86VR2
Q9C0H2
Q9NWS8
P04920
P24539
Q08722
Q86UP2
Q99442
Q9HDC5
Q9Y375
